# A Novel Eye Tracking–Based Gamified Assessment of Contrast Sensitivity Function in Children: Prospective Development and Reliability Study

**DOI:** 10.2196/81082

**Published:** 2025-11-20

**Authors:** Yunsi He, Yijing Zhuang, Lei Feng, Xuanyu Xu, Ying Deng, Moruomi Li, Yangfei Pang, Ying Yao, Wentong Yu, Zixuan Xu, Yusong Zhou, Yudan Zhong, Qiuying Li, Qingqing Ye, Junpeng Yuan, Yun Wen, Jinrong Li

**Affiliations:** 1 State Key Laboratory of Ophthalmology, Zhongshan Ophthalmic Center, Sun Yat-sen University, Guangdong Provincial Key Laboratory of Ophthalmology and Visual Science, Guangdong Provincial Clinical Research Center for Ocular Diseases, 54 Xianlie South Road, Guangzhou, 510060, China, 86 020 8733 0351; 2 Lightspeed Studios, L&Q Oasis PTE. Ltd, Singapore, Singapore

**Keywords:** pediatric vision assessment, contrast sensitivity function, eye tracking, gamified testing, test–retest reliability

## Abstract

**Background:**

Reliable assessment of visual function in young children remains a challenge. Contrast sensitivity function (CSF) is a sensitive and fundamental index of visual performance, yet existing pediatric CSF assessments lack objectivity and adaptability. To bridge this methodological gap, we developed a novel eye tracking–based gamified contrast sensitivity function (ETGCSF) tool that integrates gaze-based detection with interactive gameplay to objectively quantify CSF in an engaging and child-centered manner.

**Objective:**

This study aimed to (1) establish the feasibility and test-retest reliability of the ETGCSF tool in preschool-aged children and (2) evaluate whether optimization using adaptive algorithms and enhanced gamification elements could improve test efficiency while maintaining reliability.

**Methods:**

This was a prospective study with 2 sequential cohorts. A total of 80 Chinese children aged 3 to 6 years were pragmatically recruited from Zhongshan Ophthalmic Center between May 2021 and July 2023. On the basis of timing of data collection, 35% (28/80) of the children were included in experiment 1 (mean age 5.24, SD 0.15 years), and 65% (52/80) were included in experiment 2 (mean age 4.76, SD 0.11 years). Children completed 2 runs of ETGCSF test. Experiment 1 used the baseline ETGCSF protocol, and experiment 2 used the optimized protocol. Primary outcomes were test-retest reliability of the area under the log contrast sensitivity function curve (AULCSF) and CSF acuity, reported as intraclass correlation coefficients (ICCs) with 95% CIs.

**Results:**

In experiment 1, the ETGCSF tool showed strong reliability, with ICCs of 0.890 (95% CI 0.741‐0.951) for AULCSF and 0.890 (95% CI 0.763‐0.949) for CSF acuity. The median test duration was 482 (IQR 451‐569) seconds. In experiment 2, the optimized ETGCSF reduced median test duration to 241 (IQR 189‐296) seconds (*P*<.001) while maintaining comparable reliability. AULCSF estimates varied by 0.03 log units across 2 runs (95% CI −0.51 to 0.57; *t*_51_=0.749; *P*=.46), with an ICC of 0.851 (95% CI 0.740‐0.914; *P*<.001) that was not significantly different from that of experiment 1 (*z*=0.660; *P*=.51). Similarly, CSF acuity estimates varied by 0.004 log units (95% CI –0.33 to 0.32; *t*_51_=0.192; *P*=.85), with an ICC of 0.832 (95% CI 0.708‐0.904; *P*<.001), also comparable to that of experiment 1 (*z*=–0.925; *P*=.36).

**Conclusions:**

This study introduces a paradigm shift in pediatric visual assessment by leveraging objective eye tracking and gamified engagement to transform contrast sensitivity testing into a scalable, child-friendly process. The ETGCSF tool demonstrated strong reliability and markedly improved efficiency in assessing CSF in preschool children aged 3 to 6 years. These findings support ETGCSF as a promising tool for real-world clinical practice, and its modular design holds potential for future adaptations ranging from streamlined rapid screening in very young children to full CSF profiling for research.

## Introduction

The early years of life represent a critical period for visual development during which appropriate visual input is essential to ensure proper maturation of the visual system [1]. Atypical visual experiences during this stage can lead to long-term or even permanent vision impairments if left unaddressed. Therefore, early detection and timely intervention are crucial in mitigating the impact of pathological visual deprivation and promoting optimal visual outcomes. Vision assessment during this sensitive period plays a pivotal role in the prevention and treatment of visual impairments, offering a window of opportunity to prevent lifelong consequences [2].

Despite recent advances in pediatric vision assessment techniques, significant challenges remain in evaluating visual function in young children. Current methods face limitations stemming from 2 primary factors: the cooperation abilities of young children and the inherent constraints of existing assessment tools. Children often struggle to engage with tests that require subjective feedback or behavioral responses, such as verbal descriptions or manual pointing. To address this, techniques such as preferential looking [3] rely on examiners observing subtle indicators, including eye and head movements, facial expressions, and nonverbal gestures, to infer visual responses [4]. More recently, new contrast sensitivity tests such as the Double Happy test, a preferential looking–based contrast sensitivity assessment, have been developed to incorporate fun stimuli to appeal to children [5]. However, these methods are inherently subjective, with outcomes influenced by the examiner’s experience and judgment, leading to potential variability and bias. Furthermore, many current assessment tools focus predominantly on high-contrast visual stimuli, such as stripes or patterns, which only assess a narrow range of visual function. This lack of comprehensive evaluation limits the ability to detect subtle but clinically significant deficits in visual performance, particularly in low-contrast environments or other essential aspects of visual development. These challenges underscore the urgent need for innovative, objective, and reliable assessment approaches capable of overcoming these limitations and providing a more accurate assessment of early childhood visual function.

Among various measures of spatial vision, the contrast sensitivity function (CSF) stands out for its ability to provide a comprehensive assessment of spatial vision and detect subtle visual deficits [6]. CSF is particularly valuable in diagnosing ocular diseases in which early visual impairments may not be detectable through standard methods, and it also plays a crucial role in monitoring disease progression and assessing treatment efficacy [7,8]. Widely used in both clinical and research settings, CSF is traditionally measured using chart-based [9,10] or computer-based psychophysical methods [11-13]. Recent advancements in infrared eye tracking technology have further enhanced the potential of CSF assessment by enabling the use of eye movements as objective, nonverbal indicators of visual function. By monitoring different aspects of eye movement patterns, such as fixation [4,14-18], microsaccades [19], saccades [20-22], smooth pursuit [23,24], and optokinetic nystagmus [25,26], the detection or discrimination threshold to the visual stimulus can be estimated objectively and precisely. The literature indicates that eye tracking can quantify pediatric detection thresholds at specific spatial frequencies (SFs), and several studies have further derived full CSF profiles, highlighting its potential for pediatric CSF assessment.

Another major challenge in contrast sensitivity testing for children is the prolonged testing time and the associated risk of attention lapses, particularly during repeated exposure to the same pattern. As a psychophysical assessment, the reliability of contrast sensitivity measurements is highly dependent on children’s sustained attention and cooperation. Lapses typically underestimate true visual ability [27,28]. Incorporating gamified elements into the testing process can help mitigate this problem by enhancing test appeal and increasing children’s interest and engagement [29]. For example, a tablet-based interactive “pop CSF” game was developed in which children touched the screen to “catch” a Gabor patch; this approach successfully estimated CSF and differentiated visual impairment in children [30,31], underscoring the promise of gamification for pediatric CSF assessment. However, because touch-based paradigms rely on overt motor responses, they are prone to response bias and attentional lapses and may be systematically confounded by eye-hand coordination demands, potentially diluting the validity of the CSF estimate.

Building on this evidence, our preliminary studies have demonstrated the feasibility of integrating gaze position recording with the preferential looking technique to objectively quantify CSF, offering a rapid, automated, and reliable approach to measuring spatial vision in adults [32]. Nonetheless, translating these gains to testing in preschool-aged children requires further optimization. In particular, it is necessary to shorten test time, sustain engagement, and minimize the need for verbal or manual responses given the limited cooperation and attention span of young children [29,30,33]. These considerations motivate this work, which targets an objective, child-centered CSF paradigm designed for efficiency, engagement, and robustness in real-world pediatric settings.

In this study, we introduced a novel eye tracking–based gamified contrast sensitivity function (ETGCSF) assessment designed to mitigate the challenges of pediatric vision assessment. The objective of this study was to develop and validate the ETGCSF test for children by (1) assessing its feasibility and reliability and (2) determining the impact of optimization on its efficiency, reliability, and user engagement. This approach aimed to balance these factors, providing a robust foundation for its application in clinical and real-world pediatric vision assessments.

## Methods

### Ethical Considerations

This study adhered to the principles of the Declaration of Helsinki and was reviewed and approved by the institutional ethics committee of the Zhongshan Ophthalmic Center, Sun Yat-sen University (approval 2023KYPJ343). Written informed consent was obtained from the parents or legal guardians of all participants before study enrollment. All data were anonymized before analysis, and no personally identifiable information was retained in the research dataset to ensure participant privacy and confidentiality. Participants did not receive financial compensation. No images or supplementary materials that could reveal the identity of participants are included in this manuscript.

### Participants

This was a prospective development and reliability study with 2 sequential cohorts. Participants were recruited via clinic-based pragmatic sampling during routine outpatient visits from the visual rehabilitation clinic at the Zhongshan Ophthalmic Center, a tertiary ophthalmic hospital in Guangzhou, China, between May 2021 and July 2023. The sample size was determined pragmatically based on the number of eligible children available, clinic flow, and staff availability during the study period. Inclusion criteria were age of >3 and <6 years, uncorrected visual acuity (UCVA) better than 1.0 logarithm of minimum angle of resolution (logMAR), and parental or guardian consent to participate. Exclusion criteria were blepharoptosis or nystagmus given their potential to interfere with accurate eye tracking. Participation was voluntary, and refusal to participate did not affect the children’s ongoing clinical care.

Distance UCVA was measured using a Tumbling E Early Treatment Diabetic Retinopathy Study chart (Wehen Vision) illuminated to a luminance of 200 cd/m^2^ and viewed from 4 meters. Visual acuity was recorded in logMAR units, with each correctly identified letter contributing to the score.

A total of 80 Chinese children aged 3 to 6 years were included in the study. Of the 80 participants, 28 (35%; mean age 5.24, SD 0.15 years; n=12, 43% male) were assigned to experiment 1, and 52 (65%; mean age 4.76, SD 0.11 years; n=24, 46% male) were assigned to experiment 2 based on the timing of data collection. Experiment 1 assessed the feasibility and test-retest reliability of the ETGCSF tool to establish its baseline performance in young children. Experiment 2 built on these findings by incorporating enhanced gamified elements such as cartoon characters, adaptive feedback, and fewer trials to improve test efficiency and participant engagement. Examiners completed protocol training before testing. Each participant underwent 2 consecutive ETGCSF tests on the same eye to evaluate within-session variability. Each measurement was conducted independently, with both examiners and participants masked to previous results.

### Equipment and Stimuli

Gaze position was recorded using a Tobii Pro Nano remote eye tracker (Tobii AB) with a sampling rate of 65 Hz. Visual stimuli were displayed on a 27-inch Republic of Gamers Swift PG278QR monitor (ASUSTeK Computer Inc) with a resolution of 2560 × 1440, a refresh rate of 165 Hz, and a luminance of 52.1 (SD 1.3) cd/m^2^. Real-time gaze monitoring was conducted using a 14-inch Lenovo Xiaoxin Air-14 IIL desktop computer (2020 model).

**Figure 1.**
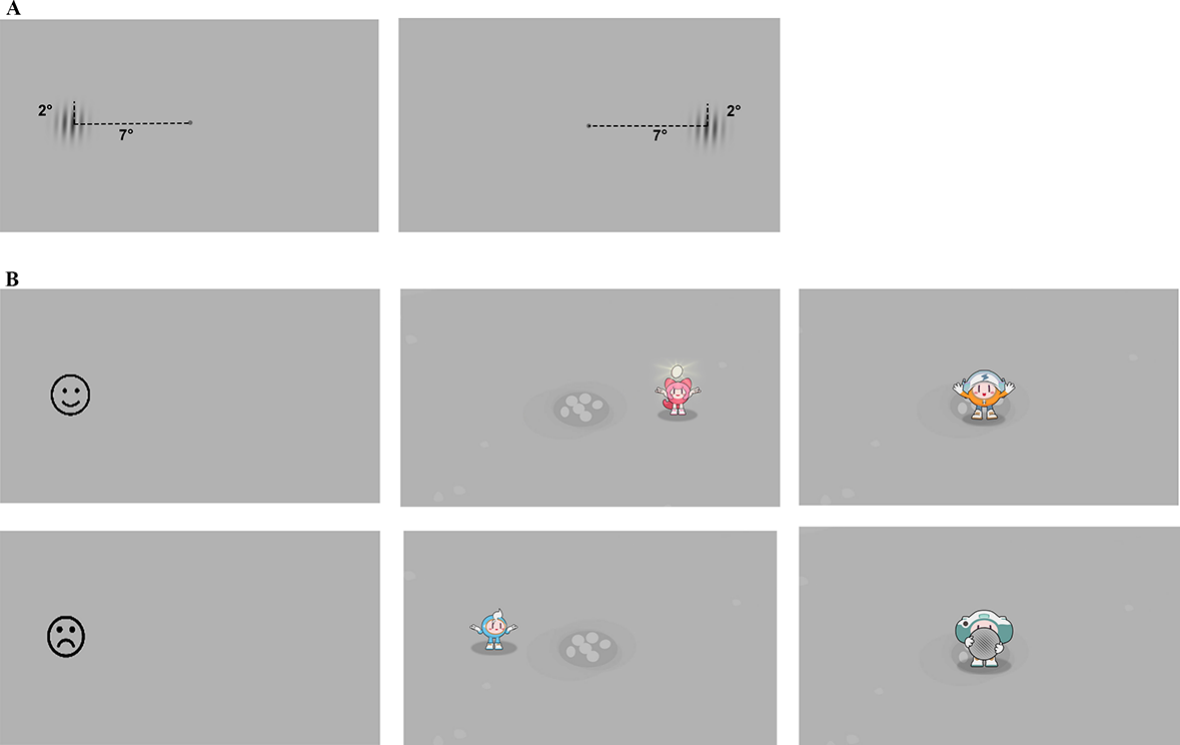
Visual stimulus and feedback designs for the eye tracking–based gamified contrast sensitivity function test from a prospective study on its development and reliability. This study was conducted among preschool children (aged 3‐6 years) at the Zhongshan Ophthalmic Center in Guangzhou, China, between May 2021 and July 2023. (A) Schematic showing the location of Gabor patches, positioned 7 degrees to the left or right of the central fixation point. (B) Evolution of feedback designs: the stick figures used in the initial protocol (experiment 1, left) were replaced with gamified animations featuring the Quantum Family in the optimized protocol (experiment 2, middle and right).

The primary visual stimulus was a Gabor patch with a radius of 2 degrees of visual angle. To minimize detection cues, the edges were blurred using a half-Gaussian ramp spanning 0.5 degrees of visual angle. The orientation of the patches was randomized between 0 and 180 degrees. Both the contrast and SF were dynamically adjusted using an adaptive algorithm. In each trial, a Gabor patch was randomly displayed on the left or right side of the monitor, positioned 7 degrees from the center (Figure 1A).

### Interactive Feedback Design

To maintain engagement, experiment 1 used feedback stimuli consisting of blinking fruit-shaped stick figures at the center of the screen to capture attention and ensure central fixation. For correct responses, a smiling face was displayed, whereas incorrect responses triggered a crying face (Figure 1B, left panel).

Building on these insights, experiment 2 introduced gamified feedback designed in collaboration with the renowned game company LightSpeed Studios. On the basis of the validation of our feasible method in experiment 1, we further incorporated game elements into the design, with the Quantum Family cartoon characters integrated to make the task more vivid and engaging. At the start of each trial, the character greeted the child with a friendly “Hi” (attention cue). Feedback was tailored to responses: correct answers triggered an animation of the character digging up a turtle egg with praise, whereas incorrect answers prompted encouraging gestures. Additional elements included a bonus cue for 3 consecutive correct responses (eg, enthusiastic greetings) and an instruction cue to re-explain the task after 3 consecutive incorrect responses (Figure 1B, middle and right panels).

### CSF Module and Eye Tracking Integration

**Figure 2.**
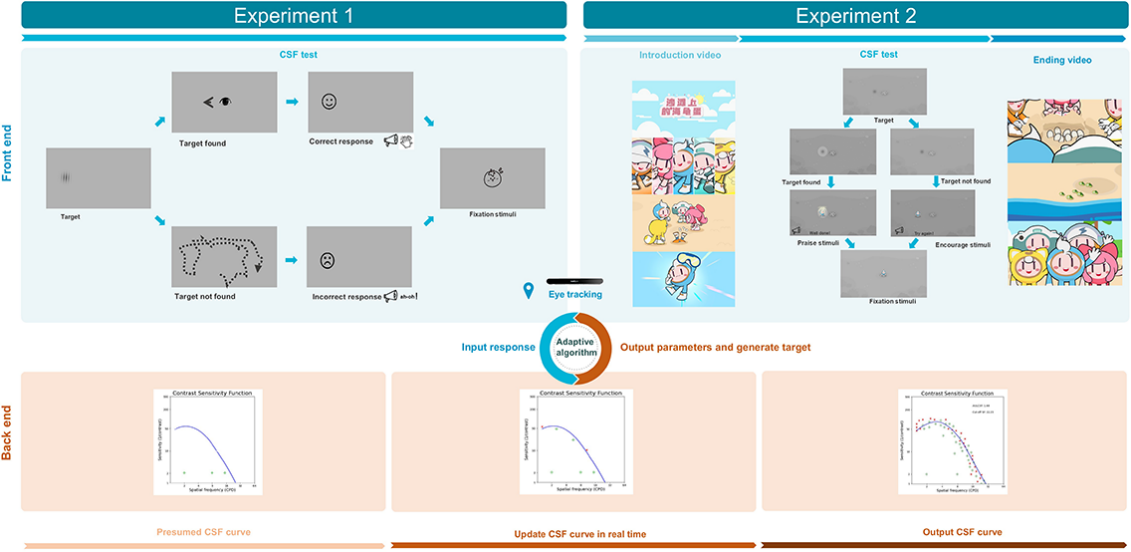
Testing procedure and data processing pipeline for the eye tracking–based gamified contrast sensitivity function (CSF) test. This figure illustrates the workflow for both the baseline (experiment 1) and optimized (experiment 2) protocols from a prospective development and reliability study. This study was conducted among preschool children (aged 3‐6 years) attending a visual rehabilitation clinic at the Zhongshan Ophthalmic Center, Guangzhou, China (May 2021-July 2023). The system engaged children via a front-end interface, monitored gaze accuracy through an eye tracking module, and dynamically updated stimulus parameters and the CSF curve via a back-end algorithm.

The test program comprised 2 interconnected modules: the CSF module and the eye tracking module (Figure 2). The CSF module used the quick contrast sensitivity function (qCSF) algorithm, a Bayesian adaptive procedure developed by Lesmes et al [34], to iteratively update 4 key parameters of the CSF curve: peak sensitivity (*γ*_*max*_), peak SF (*f*_*max*_), bandwidth (β), and truncation on the low-frequency side (δ). These parameters modeled the CSF as a truncated log-parabola, capturing individual variations in contrast sensitivity across SFs. By integrating previous knowledge of the CSF’s general shape with trial-by-trial data, the qCSF algorithm selected the optimal combination of contrast and SF at each trial to maximize information gain, improving test efficiency while maintaining reliability and precision. Upon test completion, the algorithm generated a complete CSF curve and computed 2 critical metrics: the area under the log contrast sensitivity function curve (AULCSF) and CSF acuity.

The eye tracking module monitored gaze position in real time to determine response accuracy based on fixation behavior. A correct response was defined as maintaining gaze within a rectangular area centered on the stimulus (4.4 degrees of visual angle) for at least 0.8 seconds, whereas gaze outside this area for more than 4 seconds indicated an incorrect response. After each trial, the eye tracking module communicated the response accuracy to the CSF module, which then adjusted the contrast and SF of the target stimulus for the next trial to maximize information gain. This real-time integration ensured efficient and adaptive testing.

### Refinements in Testing Parameters

In experiment 1, the CSF module required 60 trials per session to generate a complete CSF curve, with the SF of the target stimuli ranging from 0.5 to 32 cycles per degree (cpd). Building on these findings, experiment 2 introduced refinements to improve testing efficiency and better accommodate pediatric participants. Enhancements to the Bayesian adaptive algorithm reduced the number of trials to 30 per session while maintaining reliability and precision. Additionally, the SF range was adjusted to 0.5 to 15 cpd to reflect the developmental visual characteristics of young children.

### Testing Setup and Experimental Protocols

#### General Testing Setup and Monitoring

Testing was conducted in a dimly lit room, with children seated either alone or on a parent’s lap at a viewing distance of 60 cm. Each eye was tested monocularly, with the other eye patched. Before testing, a 2-point calibration was performed using the Tobii Pro Nano’s built-in calibration program to ensure the accuracy of eye tracking. Real-time gaze positioning was monitored throughout the test, enabling the examiner to detect significant deviations in head position or signs of distraction. Adjustments were made as needed, and the test was terminated if necessary to ensure reliable data collection.

#### Experiment 1: Standard Testing Procedure

In experiment 1, each trial began with a fruit-shaped stick figure displayed at the center of the screen to capture the child’s attention, followed by the appearance of the Gabor patch target. Feedback was provided based on the child’s response: a smiling face for correct responses or a crying face for incorrect responses, signaling the transition to the next trial. Each session consisted of 60 trials, after which the program generated a complete CSF curve (Figure 2, bottom left panel).

#### Experiment 2: Gamified Testing Procedure

In experiment 2, enhanced gamification elements were introduced to improve participant engagement. The session began with an introductory video featuring the Quantum Family cartoon characters, who accidentally scattered turtle eggs on a beach. The narrative introduced a magical pair of glasses that transformed the beach into a grayscale image, revealing the striped cloth (representing the Gabor patch) as the hidden location of the eggs. Children were invited to locate the eggs and return them to the nest.

After this, an instructional video explained how to identify the Gabor patch. Each trial began with an attention cue followed by the target presentation. On the basis of the child’s response, a reward cue (eg, praise animations) or a motivation cue (eg, encouraging gestures) was displayed to transition to the next trial. Each session consisted of 30 trials. The session concluded with a video showing the Quantum Family returning the eggs to the nest, where they hatched into turtles and swam back to the sea. A CSF curve was generated and displayed at the end of the session (Figure 2, bottom right panel).

The Gabor patch was presented within a predefined target area on a uniform grayscale background, and no other patterns were present within this region, ensuring that gaze responses reflected detection of the Gabor stimulus itself.

### Statistical Analysis

The primary CSF metrics included the AULCSF and CSF acuity. AULCSF, calculated by integrating the fitted CSF curve across SFs from 1.5 to 18 cpd, reflected overall spatial vision function. Notably, the fitted curve could extend beyond the maximum stimulus frequency actually presented. CSF acuity was defined as the logMAR-equivalent value corresponding to the SF at a 100% contrast threshold, representing the ability to discern fine details under medium to high contrast conditions. This conversion provided a pragmatic means of situating the psychophysical results within a clinically familiar framework.

Statistical analyses were conducted using SPSS (version 26.0; IBM Corp). Continuous variables with normal distributions were expressed as means and SDs, whereas nonnormally distributed variables were reported as medians with IQRs. The Spearman rank correlation coefficient was used to analyze relationships between test duration and age. Test reliability was assessed using the intraclass correlation coefficient (ICC) [35]; Bland-Altman analysis [36]; and the coefficient of repeatability (CoR), with lower CoR values indicating greater consistency. ICCs were compared across methods using the Fisher *Z* transformation to test for significant differences. Fractional rank precision (FRP) was calculated to assess the test’s ability to distinguish participants based on their scores, with higher FRP values (closer to 1.0) indicating greater test-retest consistency [37]. A *P* value of less than .05 was considered statistically significant. Results were reported in compliance with the Guidelines for Reporting Reliability and Agreement Studies criteria [38].

## Results

### Experiment 1

**Figure 3.**
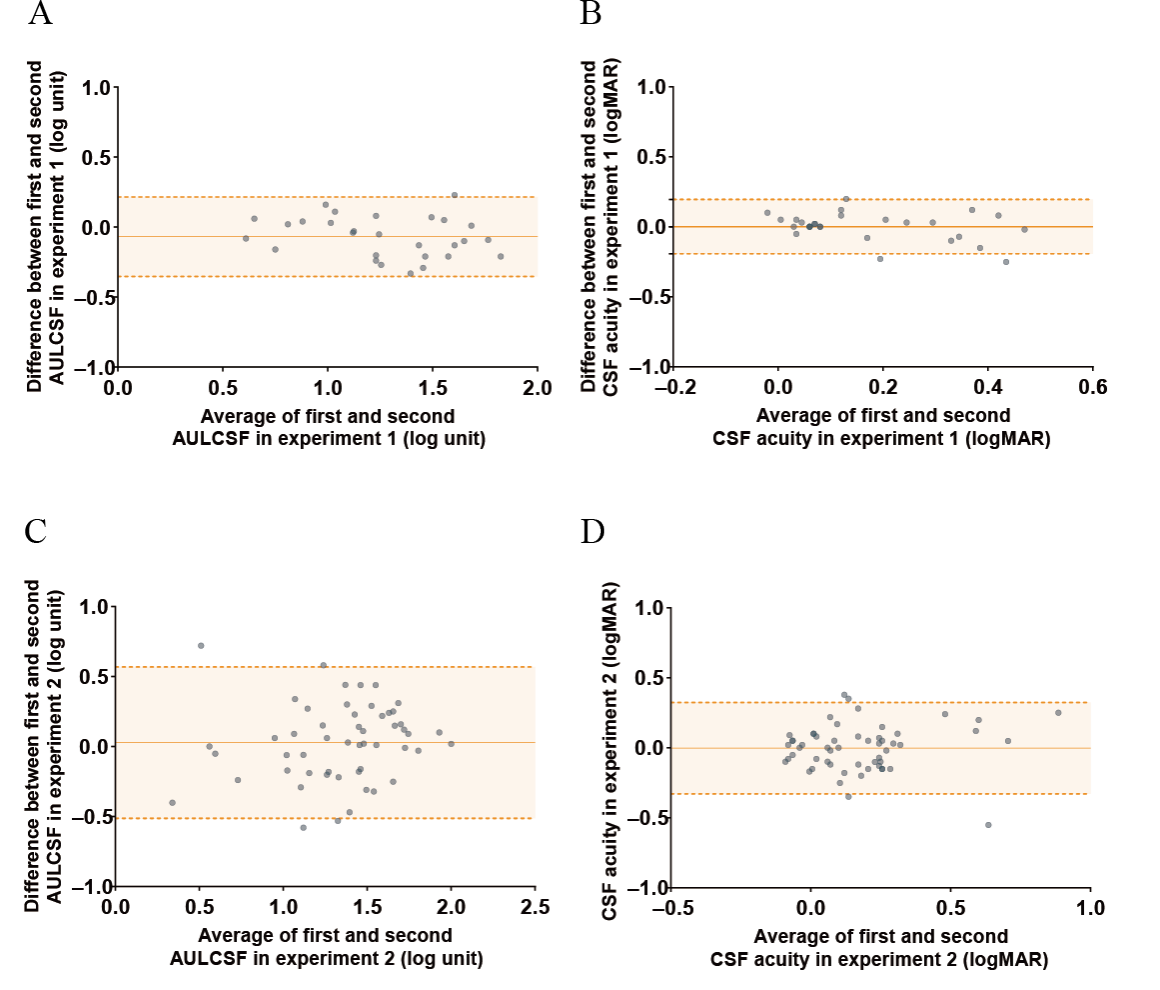
Test-retest agreement of the eye tracking–based gamified contrast sensitivity function (CSF) measured using Bland-Altman analysis. This analysis was part of a prospective study conducted among preschool children (aged 3‐6 years) at the Zhongshan Ophthalmic Center, Guangzhou, China (May 2021-July 2023). Plots display the difference between test and retest measurements against their mean for the area under the log contrast sensitivity function (AULCSF) and CSF acuity in the baseline protocol (A and B) and the optimized protocol (C and D). In each plot, the central solid orange line represents the mean difference (bias), and the top and bottom dotted orange lines indicate the upper and lower limits of the 95% agreement interval. Individual data points are shown as semitransparent green circles, with darker shading indicating overlapping values. logMAR: logarithm of minimum angle of resolution.

All 28 children successfully completed 2 rounds of ETGCSF testing in the same eye. The mean UCVA was 0.33 (SD 0.04) logMAR. The median test duration was 482 (IQR 451-569) seconds. Test duration was not correlated with age (Spearman ρ=−0.006; *P*=.98). A mild learning effect was observed in the AULCSF, with estimates varying by a mean of 0.07 log units across 2 runs (95% CI −0.35 to 0.22; *t*_27_=2.755; *P*=.01; Figure 3A), with a CoR of 0.319. The ICC for the AULCSF was 0.890 (95% CI 0.741‐0.951; *P*<.001), and the FRP was 0.80. For CSF acuity, no learning effect was observed, with estimates varying by 0.001 log units across 2 runs (95% CI −0.19 to 0.19; *t*_27_=0.057; *P*=.96; not significant; Figure 3B), with a CoR of 0.194. The ICC for CSF acuity was 0.890 (95% CI 0.763‐0.949; *P*<.001), and the FRP was 0.79.

### Experiment 2

All 52 children successfully completed 2 ETGCSF runs in the same eye. The mean UCVA was 0.37 (SD 0.17) logMAR. The median test duration was 241 (IQR 189-296) seconds, significantly shorter than in experiment 1 (*z*=–7.344; *P*<.001). Test time was not associated with age (Spearman ρ=0.056; *P*=.74). No learning effect was observed between initial and retest measurements for either AULCSF or CSF acuity. AULCSF estimates varied by 0.03 log units across 2 runs (95% CI −0.51 to 0.57; *t*_51_=0.749; *P*=.46; not significant; Figure 3C), with a CoR of 0.538. The ICC for the AULCSF was 0.851 (95% CI 0.740‐0.914; *P*<.001), closely matching the value from experiment 1 (*z*=0.660; *P*=.51). The FRP was 0.82. Similarly, CSF acuity estimates varied by 0.004 log units (95% CI −0.33 to 0.32; *t*_51_=0.192; *P*=.85; not significant; Figure 3D), with a CoR of 0.327. The ICC for CSF acuity was 0.832 (95% CI 0.708‐0.904; *P*<.001), which was also comparable to that observed in experiment 1 (*z*=–0.925; *P*=.36). The FRP in this case was 0.80.

## Discussion

### Principal Findings

This study evaluated whether an ETGCSF paradigm functions effectively in preschoolers and whether a more engaging, adaptive version could achieve faster testing without sacrificing reliability. In preschool children, the initial implementation was feasible and reproducible, with high test-retest reliability. The optimized version achieved substantially shorter testing time while maintaining comparable reliability. Overall, these results indicate that the ETGCSF tool provides an objective, child-friendly readout of CSF that balances precision and throughput, supporting its suitability for real-world pediatric use.

With the development of infrared eye tracking technology, eye movements have been adopted as nonverbal cues in psychophysical measurements. Building on this concept, our study incorporated eye tracking technology and further optimized the testing interface and underlying algorithm to create a child-friendly paradigm that preserves objectivity while enhancing efficiency and engagement. Cartoon feedback and gamified settings increased appeal, whereas the adaptive algorithm efficiently selected optimal stimulus parameters to balance test duration and coverage. The resulting ETGCSF paradigm is specifically tailored to the developmental and behavioral characteristics of pediatric populations. Although this study focused primarily on quantitative reliability, informal observations also suggested that the gamified interface in experiment 2 promoted higher engagement. Children appeared more attentive, with fewer interruptions or signs of fatigue during the test than in experiment 1. While these impressions were not formally measured, they support the potential of game-based strategies to improve cooperation in pediatric testing—a hypothesis that may warrant future investigation using standardized behavioral metrics or caregiver-reported engagement scales.

In addition to eye tracking and gamified design, a key design choice concerned the SF range. In experiment 1, we used a broader prior, which was comprehensive but raised occasional concerns about potential aliasing at very high frequencies. Although the adaptive algorithm seldom probed these extremes in preschoolers, this experience informed the next iteration. In response, experiment 2 narrowed the upper range to minimize distortion, shorten testing time, and align more closely with real-world pediatric needs. The finalized window of 0.5 to 15 cpd is intentional: it targets the clinically common and diagnostically challenging mild to moderate deficits, where sensitive detection can influence management. Severe impairment is typically identifiable using simpler, established tools. In this context, ETGCSF is designed to fill the gap between high-contrast acuity tests and comprehensive psychophysics by providing an objective, scalable CSF readout suited to routine pediatric workflows and adaptable to telehealth.

The ETGCSF tool further demonstrated substantial efficiency gains in experiment 2, where reductions in trial numbers shortened the median test duration compared to experiment 1. This improvement was achieved without compromising reliability or validity. The lack of a significant correlation between test duration and age should be interpreted within the context of the study’s relatively narrow age range. This finding indicates that test time remained consistent across the specific preschool cohort examined in this study. Future studies encompassing a wider age distribution are warranted to conclusively determine the influence of age on test efficiency.

This study established the reliability and feasibility of the ETGCSF tool for assessing CSF in children. Experiment 1 demonstrated excellent test-retest reliability for both AULCSF and CSF acuity [35] despite individual variability in measurements, likely due to the heterogeneous population. To facilitate clinical interpretation, CSF acuity was expressed in logMAR-equivalent units converted from the cutoff SF, providing a pragmatic bridge between psychophysical measures and conventional acuity metrics. Experiment 2 incorporated a more efficient design with a reduced trial burden, which shortened test durations and mitigated learning effects during retesting. This gain in efficiency involved a trade-off, manifesting as a slight reduction in test precision (evidenced by higher CoR values and wider limits of agreement); however, the preserved high ICCs affirm the method’s reliability for group-level analyses. Moreover, the improved rank precision in experiment 2 further underscores its robustness in distinguishing subtle changes in visual performance. Together, these findings indicate that the ETGCSF tool can balance efficiency and reliability in pediatric vision assessments. In a study using the qCSF method for repeated testing in adults with multiple sclerosis, Rosenkranz et al [39] reported a mean difference of –0.049 log units for the AULCSF with a CoR of 0.228 and a mean difference of –0.036 log units for CSF acuity with a CoR of 0.137. Finn et al [40] reported that the qCSF-derived AULCSF and CSF acuity showed excellent reliability, with a CoR of 0.16 and 0.17 log units, respectively. In comparison, the retest variability of our method was larger for both of these metrics, which is potentially attributable to poorer compliance in preschool children compared to adults. Commonly used clinical methods such as the Pelli-Robson chart, the CSV-1000, and the qCSF test typically demonstrate repeatability coefficients ranging from approximately 0.23 to 1.38 log contrast sensitivity across SFs [13,41], and the CoR in our study fell within this range. While the CoR is prone to scaling artifacts, the FRP’s strength lies in providing a fair, scale-invariant comparison of reliability between multiple tests administered to the same study population. Although FRP values cannot be directly compared across studies, the high FRP observed in our cohort indicates that our method has excellent discriminative ability and stability, effectively distinguishing individuals with better and worse visual function within the study population.

### Limitations

This study has several limitations. First, as participants were primarily recruited from a clinical setting and limited to preschool children aged 3 to 6 years, the findings may not fully reflect the tool’s performance in community-based assessment programs or in younger children; future work should include more diverse child populations, including younger children and those with varied ocular and systemic conditions. Second, we did not benchmark the ETGCSF tool against gold-standard methods such as the preferential looking protocol; direct head-to-head comparisons are needed to confirm its clinical equivalence. Third, although the gamified design appeared to improve engagement, we did not quantitatively measure compliance or attention; incorporating objective engagement metrics will clarify the true impact of gamification on test reliability.

### Future Work

Looking forward, the modular design of the ETGCSF paradigm allows for adaptation to diverse clinical and research needs. While this study validates the full CSF assessment, the platform could be streamlined for rapid screening in challenging populations (eg, very young children) by focusing on 1 or 2 critical SFs. On the other hand, the full CSF profile remains invaluable for investigating developmental trajectories and nuanced visual deficits in neuro-ophthalmic disorders. Therefore, the ETGCSF tool provides a versatile foundation for both efficient clinical screening and in-depth vision science research.

### Conclusions

This study introduces ETGCSF as an objective, efficient, and child-centered assessment of contrast sensitivity for preschool children. Its distinctive contribution lies in coupling eye tracking–derived, nonverbal readouts with gamified task flow, allowing attention to be stabilized and responses to be inferred without speech or pointing while maintaining reliability. Addressing limitations of response-dependent methods, this paradigm functions as a modular framework that can be configured for quick screening or comprehensive CSF profiling and aligned with routine clinics, community screening, and telehealth. Although validation across younger and more diverse populations and direct benchmarks against gold-standard pediatric tests are still required, the findings support practical, near-term translation and point toward broader adoption of objective, digital vision assessments.

## Abbreviations

AULCSF: area under the log contrast sensitivity function curve
CoR: coefficient of repeatability
cpd: cycles per degree
CSF: contrast sensitivity function
ETGCSF: eye tracking–based gamified contrast sensitivity function
FRP: fractional rank precision
ICC: intraclass correlation coefficient
logMAR: logarithm of minimum angle of resolution
qCSF: quick contrast sensitivity function
SF: spatial frequency
UCVA: uncorrected visual acuity
